# Physiological and genomic insights into abiotic stress of halophilic archaeon *Natrinema altunense* 4.1R isolated from a saline ecosystem of Tunisian desert

**DOI:** 10.1007/s10709-023-00182-0

**Published:** 2023-02-16

**Authors:** Afef Najjari, Ayoub Boussetta, Noha Youssef, Javier A. Linares-Pastén, Mouna Mahjoubi, Rahma Belloum, Haitham Sghaier, Ameur Cherif, Hadda Imene Ouzari

**Affiliations:** 1 Faculté des Sciences de Tunis, LR03ES03 Laboratoire de Microbiologie et Biomolécules Actives, Université Tunis El Manar, 2092 Tunis, Tunisie; 2 Department of Microbiology and Molecular Genetics, Oklahoma State University, Stillwater, OK USA; 3 Department of Biotechnology, Faculty of Engineering, Lunds Tekniska Högskola (LTH), Lund University, P. O. Box 124, 22100 Lund, Sweden; 4 University of Manouba, ISBST, LR11-ES31 BVBGR, Biotechpole Sidi Thabet, 2020, Ariana, Tunisia; 5 Laboratory “Energy and Matter for Development of Nuclear Sciences” (LR16CNSTN02), National Center for Nuclear Sciences and Technology (CNSTN), Ariana, Tunisia

**Keywords:** Haloarchaea, *Natrinema altunense*, UV-C radiation, Oxidative stress, Osmotic stress, Genomic analysis, Molecular modeling

## Abstract

Halophilic archaea are polyextremophiles with the ability to withstand fluctuations in salinity, high levels of ultraviolet radiation, and oxidative stress, allowing them to survive in a wide range of environments and making them an excellent model for astrobiological research. *Natrinema altunense* 4.1R is a halophilic archaeon isolated from the endorheic saline lake systems, Sebkhas, located in arid and semi-arid regions of Tunisia. It is an ecosystem characterized by periodic flooding from subsurface groundwater and fluctuating salinities. Here, we assess the physiological responses and genomic characterization of *N*. *altunense* 4.1R to UV-C radiation, as well as osmotic and oxidative stresses. Results showed that the 4.1R strain is able to survive up to 36% of salinity, up to 180 J/m^2^ to UV-C radiation, and at 50 mM of H_2_O_2_, a resistance profile similar to *Halobacterium salinarum*, a strain often used as UV-C resistant model. In order to understand the genetic determinants of *N*. *altunense* 4.1R survival strategy, we sequenced and analyzed its genome. Results showed multiple gene copies of osmotic stress, oxidative stress, and DNA repair response mechanisms supporting its survivability at extreme salinities and radiations. Indeed, the 3D molecular structures of seven proteins related to responses to UV-C radiation (excinucleases UvrA, UvrB, and UvrC, and photolyase), saline stress (trehalose-6-phosphate synthase OtsA and trehalose-phosphatase OtsB), and oxidative stress (superoxide dismutase SOD) were constructed by homology modeling. This study extends the abiotic stress range for the species *N. altunense* and adds to the repertoire of UV and oxidative stress resistance genes generally known from haloarchaeon.

**Supplementary Information:**

The online version contains supplementary material available at 10.1007/s10709-023-00182-0.

## Introduction

Extremely halophilic archaea (Class *Halobacteria*) thrive in arid and semi-arid saline and hypersaline environments, such as solar salterns, hypersaline marshes and saline ponds (Viver et al. 2021, 2019). They have also been isolated from low and fluctuating salinity environments (Dombrowski et al. 2019; Najjari et al. 2021; Purdy et al. 2004; Zhang et al. 2021). Organisms thriving in such ecosystems are exposed to salinity fluctuations (Mani et al. 2020; Viver et al. 2021; Youssef et al. 2014), extreme dryness (Bolhuis et al. 2006; Merino et al. 2019), fluctuating temperatures (Shukla et al. 2016), intense solar radiation (UV) (DasSarma & DasSarma 2018; Jones & Baxter 2017; Jones et al. 2018; Yamagishi et al. 2018), as well as nutritional deficiency (Robinson et al. 2015; Stan-Lotter et al. 2002). Halophilic archaea adapted to these extreme conditions through a plethora of mechanisms making them good models for understanding cellular adaptations to harsh and variable environmental conditions (P. DasSarma et al. 2020a, b; DasSarma et al. 2020a, b) and excellent candidates for astrobiological studies (Bashir et al. 2021; Mancinelli et al. 2004; Marion et al. 2003). Tunisia harbors several endorheic saline lake ecosystems Sebkhas and Chotts characterized by unstable climatic conditions, due to the periodic flooding by the subsurface ground water associated with high salt conditions during dry phases. Those located in arid and semi-arid regions constituted an important habitat in terms of biodiversity (El Hidri et al. 2013; Guesmi et al. 2013; Najjari et al. 2021; Neifar et al. 2019).

Halophilic archaea possess two main strategies of osmotic stress tolerance (Grant 2004; Viver et al. 2021; Youssef et al. 2014). The first, the “salting-in strategy”, allows the cells to accumulate intercellular KCl equal to NaCl in the surrounding environment (DasSarma & DasSarma 2018; Youssef et al. 2014). In fact, the omnipresence of K^+^ uptake systems in cell membranes, their high ion transport rate, and their potential to control this ion flux could, at first glance, make K^+^ a good candidate to be used as an osmolyte in cells. *Halobacterium* sp. NRC-1 is one of the most well-known systems. It has several genes that code for multiple active K^+^ transporters and an active Na^+^ efflux system involved in the maintenance of an intracellular ionic concentration appropriate for growth (DasSarma & DasSarma 2018; S. DasSarma et al. 2020a, b). The second strategy, the ‘‘salting-out’’, involves the uptake or de novo synthesis of organic solutes, such as sugars (sucrose and trehalose), polyols (glycerol, sorbitol, and mannitol), amino acid derivatives, and compatible solutes (glycine-betaine, ectoine, and hydroxyl ectoine), for protection against salinity stress (DasSarma et al. 2020a, b; Dombrowski et al. 2019; Najjari et al. 2021; Youssef et al. 2014). It is worth noting that a few exceptions have been observed in some haloarchaeon, such as the case of the *Halococcus hamelinensis*, which does not use the salt-in strategy as an osmoadaptive response, but instead accumulates glycine betaine, trehalose, and glutamate into the cytoplasm (Goh et al. 2011).

In addition to osmotic stress tolerance, halophilic archaea employ several tolerance mechanisms to counteract the direct and indirect damaging effects of UV-C radiation including dark DNA repair mechanisms in halophilic archaea include nucleotide excision repair (NER), mismatch repair (MMR), and homologous recombination repair (HRR), in addition to the light repair mechanism such as photoreactivation (Baliga et al. 2004; Leuko et al. 2011). NER is a universal and highly conserved system that controls chromosomal stability in cells (Hoeijmakers 2001; Morita et al. 2010; Sancar 1996). It starts by identification of the damage site, followed by slicing and removal of the damaged strand, synthesis and joining of new strands (Capes et al. 2011; Kish & DiRuggiero 2012; Zhao et al. 2006). The MMR system detects and corrects bases incorporated by error during DNA replication (Harfe & Jinks-Robertson 2000). HHR is employed by cells to repair double-strand breaks (DSBs). Generally, the pathway includes DSB recognition, excision at broken ends, binding of the recombinase, strand pairing/exchange, branch migration, and branch resolution (Cox 1991). A few previous studies reported on the mechanisms and enzymes responsible for the UVr-C resistance in the halophilic archaea, showed that the proteins UvrA, UvrB, UvrC and UvrD are essential for UVr-C induced damage repair (Baliga et al. 2004; Leuko et al. 2011).

Exposure to environmental stresses such as UV radiation, desiccation and pH fluctuation can damage cells directly through oxidative stress due to the oxidation of cellular components and the production of ROS (Reactive Oxygen Species), including superoxide radicals (HO_2_), singlet oxygen (O_2_), peroxide H_2_O_2_ and hydroxyl radicals (HO·) (Carmel-Harel & Storz 2000; Kaur et al. 2010). Halophilic archaea employ various strategies to cope with oxidative stress caused by UV irradiation. These include the use of both antioxidant enzymes, e.g., catalase (CAT), superoxide dismutase (SOD), peroxidase, and peroxiredoxins (Alscher et al. 2002; Carmel-Harel & Storz 2000; Sattin et al. 2015), as well as non-enzymatic elements such as glutathione (GSH), thioredoxins, glutaredoxine and glutathione peroxidase (Alscher et al. 2002; Carmel-Harel & Storz 2000; Sattin et al. 2015).

While mechanisms and pathways implicated in abiotic stress defense were described in detail for some Halobacteria members, very few to no studies were reported on tolerance mechanisms in the halophilic archaeon genus *Natrinema. Natrinema*, member of the family *Natrialbaceae,* was first described in the late nineties with the discovery of two species *Natrinema pallidum* and *Natrinema pellirubrum* (McGenity et al. 1998). They require at least 1.5 M with an optimum of 3.4–4.3 M of NaCl with neutral pH as optimal for growth. It includes eight known species according to List of Prokaryotic names with Standing in Nomenclature (LPSN) (Parte et al. 2020) *Natrinema altunense*, *N. ejinorense*, *N. gari*, *N. pallidum*, *N. pellirubrum*, *N. salaciae*, *N. soli* and *N. versiforme*. To date, there is a total of 16 sequenced *Natrinema* genomes in NCBI database. Here, we attempted to assess the physiological and genomic charcterisation of *N. altunense* 4.1R strain isolated from Sabkhat Ennaouel located in the arid climatic zone of southern Tunisia to UV-C radiation, oxidative stress (H_2_O_2_) and osmotic stress. Its genome was sequenced, and the genetic determinants of abiotic stress tolerance were identified. In addition, the 3D molecular structures of putative proteins implicated in UV-C radiation, osmotic and oxidative stresses were constructed by homology modeling. To our knowledge, this work provides the first report on molecular basis of *N. altunense* species responses to abiotic stress.

## Material and methods

### Strain isolation and molecular identification

The Haloarchaeal strain 4.1R, was isolated from saline water collected from Sabkhat Ennaouel, a saline system located in Sidi Bouzid governorate in south-central Tunisia (GPS: 34°23′41.6”N, 9°47′47.5”E), in December 2020. At the time of sampling, the salt pan water had a salinity of 29.5 g/L, pH of 7.48, and was 25 °C. Physicochemical characteristics of the saline water are shown in Table S1. Isolation was performed on DSM-97 medium (containing in g/l: casamino-acids, 7.5; yeast extract, 10.0; trisodium citrate, 3.0; KCl, 2.0; MgSO_4_·7H_2_O, 20.0; FeCl_2_·4H_2_O, 0.036; NaCl, 250; agar, 15 pH = 7.4) based on the serial dilution technique (Najjari et al. 2021). Identification and phylogenetic affiliation of strain 4.1R isolate were based on 16S rRNA gene sequencing. DNA extraction was performed as previously described (Dyall-Smith 2008). 16S rRNA gene amplification and sequencing were performed using the universal archaeal primers 04F (5’-TCCGGTTGATCCTGCRG-3’) and 1492R (5’-GGTTACCTTGTTACGACTT3-’) (Lane 1991). The PCR reaction mixture, containing PCR buffer (1X), MgCl_2_ (1.5 mM), 0.25 mM of each dNTP, 0.5 µM of each primer, 0.1 µg of chromosomal DNA, and 1 U of Taq DNA polymerase (Fermentas), was used to in 50 µl to perform PCR reactions programmed as follows: 95 °C for 5 min; 35 cycles of [94 °C 45 s, 64 °C 45 s and 72 °C 1 min], and a final extension step at 72 °C for 10 min. PCR products were purified using QIAquick PCR Purification (Qiagen) kit and the clean product was Sanger sequenced with an automated capillary ABI Biosystem 3130 (Laboratory of Microorganisms and Active Biomolecules, Faculty of Sciences of Tunisia). The 16S rRNA gene sequence obtained was compared to sequences deposited EzBioCloud server (Yoon et al. 2017), and also used for phylogenetic analysis. Sequence for the 16S rRNA gene was deposited in GenBank under the accession number MW534742.1. For phylogenetic assessment, multiple sequence alignment of the obtained 16S rRNA gene sequence with closest relatives was conducted using ClustalW (Thompson et al. 1994), and the alignment was used to construct Neighbor joining phylogenetic tree in MEGAX v10.2.6 (Kumar et al. 2016). The topology was evaluated by bootstrap sampling expressed as percentage of 500 replicates (Cheng et al. 2017).

### Tolerance to salinity, pH, and temperature

The growth conditions were determined by cultivating *N*. *altunense* 4.1R (spotting 20 µl of culture) on DSM-97 agar plates for 10–20 days. The optimal temperature was determined by incubating the cells at 25, 30, 37, 40, 55, and 60 °C, keeping the pH 7.4. The optimal pH was determined at 5.0, 6.0, 8.0, 9.0, 10.0, and 11.0, incubating the cells at 37 °C. The salinity- effect was assessed at 0, 5, 7, 8, 10, 15, 20, 25, 30, and 36% w/v NaCl, growing the cells at pH 7.4 and 37 °C.

### Tolerance to UV-C irradiation

UV-C radiation tolerance was measured according to the method of Baliga (Baliga et al. 2004). *N. altunense* 4.1R strain was in optimal growth conditions in DSM-97 medium (25% NaCl) at 42 °C until early exponential phase cultures (OD 0.8), then 1 ml of cell suspension was irradiated in the dark (254 nm, 1.60 J/s/m^2^) on ice at 36, 72, 108 and 216 J/m^2^. Cells were then left to recover at 42 °C both in light and dark conditions. A non-irradiated control was processed identically. Cell viability was determined by counting colonies on DSM-97 agar plates after incubation at 42 °C for 15−30 days. *Escherichia coli* DH5 α was used as sensitive control and was grown on Bertani (LB) medium (at 37 °C). At least three independent measurements were conducted for determining radiation resistance profiles.

### Tolerance to hydrogen peroxide-induced oxidative stress

We used the method described by Kaur and his colleagues (Kaur et al. 2010) to assess the survival of isolate following exposure to H_2_O_2_. Cells from mid-log phase were diluted in isotonic saline water (25% NaCl) for a final concentration of 10^–1^ to 10^–6^ cells/ml. Dilutions were spread on DSM-97 agar plates containing different concentrations of H_2_O_2_ (5, 10, 15, 20, 25, 30, 40, 50 mM). Colonies were counted after incubation for 1–3 weeks at 42 °C, and numbers were compared to control plates without H_2_O_2_. Three independent biological replicates were performed for each treatment.

### Genome sequencing, assembly, and annotation

Genomic DNA was extracted using the MagNA Pure LC DNA isolation kit III (Roche). Whole genome sequencing was performed on Illumina HiSeq 2000 platform using MiSeq V3 kit (600 cycle) using the services of Inqaba Biotechnical Industries. Adaptor sequence removal, trimming, error correction, and assembly were performed using SPAdes (Bankevich et al. 2012), Velvet assembler (Zerbino & Birney 2008) and the A5-miseq pipeline (Tritt et al. 2012) using default settings. The quality of the assembled genome was assessed using QUAST (Gurevich et al. 2013) and the contigs were ordered using CONTIGuator v2.3 (Galardini et al. 2011). Genome annotation was performed with NCBI´s Prokaryotic Genome Annotation Pipeline (PGAP) (Tatusova et al. 2016), Rapid Annotations using Subsystems Technology (RAST) database (Aziz et al. 2008), and Integrated Microbial Genomes/Expert Review (IMG/ER) (Markowitz et al. 2012), Transfer, and ribosomal RNA genes were predicted by tRNAScan-SE software (Lowe & Eddy 1997) and RNAmmer, respectively (Lagesen et al. 2007). Predicted genes were functionally characterized using the COG (Cluster of Orthologous Genes), orthology assignment by eggNOG-Mapper tool (Huerta-Cepas et al. 2017), and KEGG (Kyoto Encyclopedia of Genes and Genomes) pathway reconstruction server (Blin et al. 2013; Moriya et al. 2007).

Whole Genome Shotgun project has been deposited at DDBJ/ENA/GenBank under the accession SHMR00000000. The version described in this paper is version SHMR01000000.

### Comparative genomic analysis

Genome sequences of closely related species were obtained from the NCBI database (http://www.ncbi.nlm.nih.gov) under GenBank accession numbers NZ_JNCS00000000.1 (*N. altunense* AJ2), JXAN00000000.1 (*N. altunense* strain:1A4-DGR) and NZ_AOIK00000000.1 (*N. altunense* JCM 12,890). In silico DNA-DNA hybridization (DDH) values were calculated by the Genome-to-Genome Distance (GGDC 2.1, http://ggdc.dsmz.de/) using the BLAST + method (http://ggdc.dsmz.de/distcalc2.php; Auch et al. 2010; Meier-Kolthoff et al. 2013) and recommended formula 2. Average Nucleotide Identity (ANI) was calculated using best hits and reciprocal best hits as described by (Lee 2008). OrthoVenn was used to identify all of the orthologous proteins between the obtained genome and the above reference genomes (Wang et al. 2015). The tool uses DIAMOND algorithm (Buchfink et al. 2015) to perform all-against-all sequence comparison and OrthoMCL (Li et al. 2003) to identify orthologous genes based on comparison with default parameters (e-value = 1e-2, inflation value = 1.5, enabled annotation and protein similarity network and disabled cluster relationships).

### In silico prediction of protein–protein interactions implicated in abiotic stress

Protein association network was assessed by subjecting the putative proteins implicated UVr-C, osmotic stress and oxidative stresses (Tables S4, S5, S6) to the String database at high confidence score (≥ 0.7) (Szklarczyk et al. 2015). This database includes direct (physical) and indirect (functional) associations through a computational forecast. The interaction networks were generated based on the *N. altunense* AJ2 genome sequence.

### Molecular modeling of putative proteins involved in abiotic stress tolerance

Putative proteins involved in responses to UV-C radiation, osmotic, and oxidative stress, were selected for molecular structure modelling. Excinucleases UvrA (WP_130169190.1), UvrB (WP_1301696694.1), and UvrC (WP_130169688.1); photolyase (WP_130169120.1), trehalose-6-phosphate synthase OtsA (WP_130171241.1), trehalose-phosphatase OtsB (WP_130171242.1), and superoxide dismutase SOD (WP_007109425.1) were modelled. The 3D structures were built using YASARA software (Krieger & Vriend 2014), using several templates for each model, in order to get hybrid models. The structures were analyzed and depicted with Chimera (Pettersen et al. 2004). Signal peptides were analyzed with Signal IP v4.1 (Nielsen 2017).

## Results

### Identification and phylogenetic assignment of 4.1R strain

**Fig. 1:**
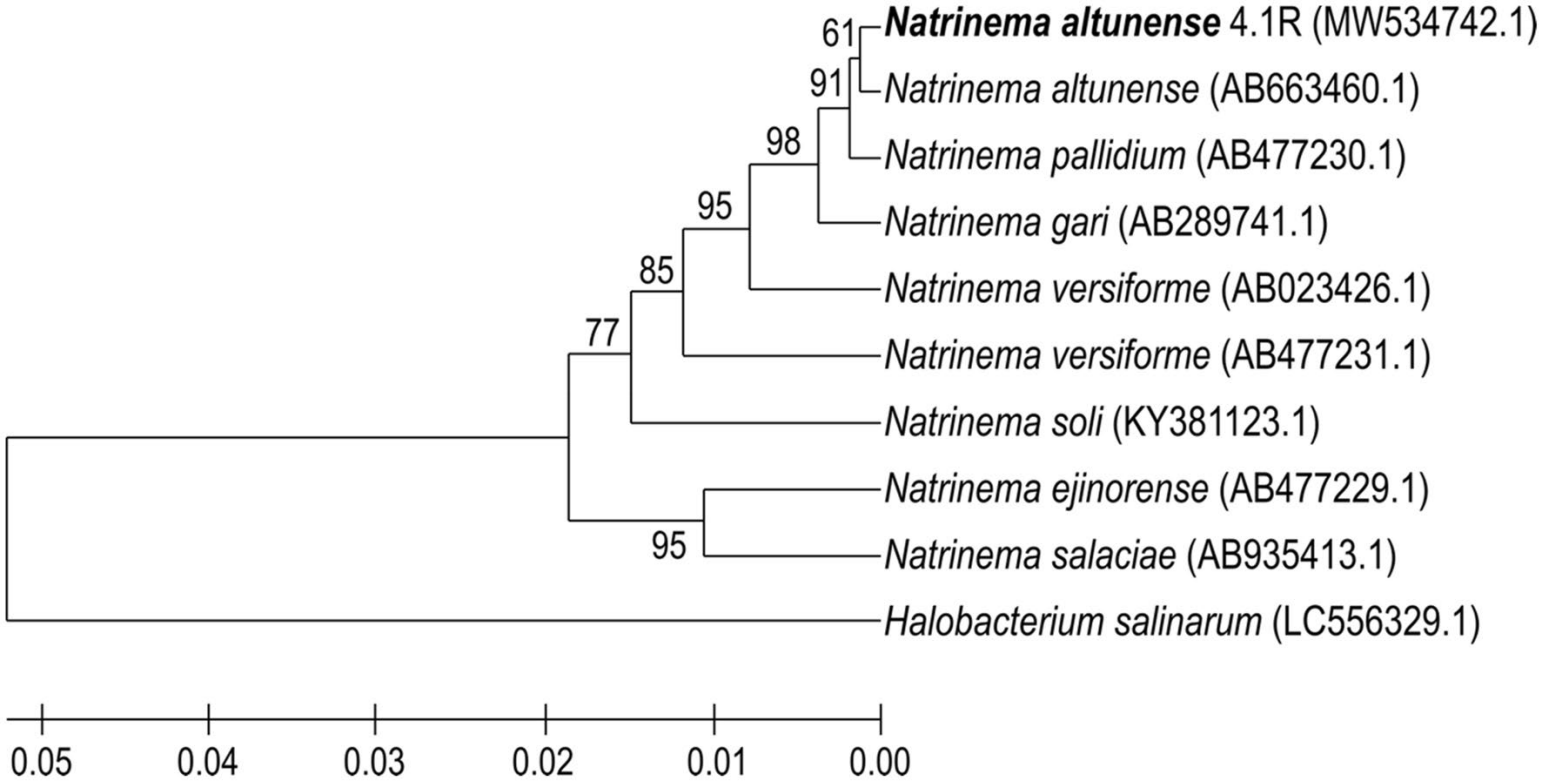
Phylogenetic tree of *N. altunense* 4.1R strain with its closest relative species based on 16S rRNA gene sequences. The evolutionary history was inferred using the UPGMA method. The percentage of replicate trees in which the associated taxa clustered together in the bootstrap test (500 replicates) are shown next to the branches. The tree was rooted with *Halobacterium salinarum* (LC556329.1). Evolutionary analyses were conducted in MEGAX v10.2.6. GenBank Accession number of sequences are shown in parenthesis

In the agar plate 4.1R colonies have a red color and cells are rod-shaped (Fig. S1). Results of 16S rDNA gene sequencing and phylogenetic assignment to the closest related species showed that 4.1R strain is most close to *N. altunense* AJ2 (NR_112855.1) with 99.68% of similarity (Fig. 1).

### Physiological tolerance of the obtained isolates to abiotic stress

#### Tolerance to salinity, pH, and temperature

**Fig. 2:**
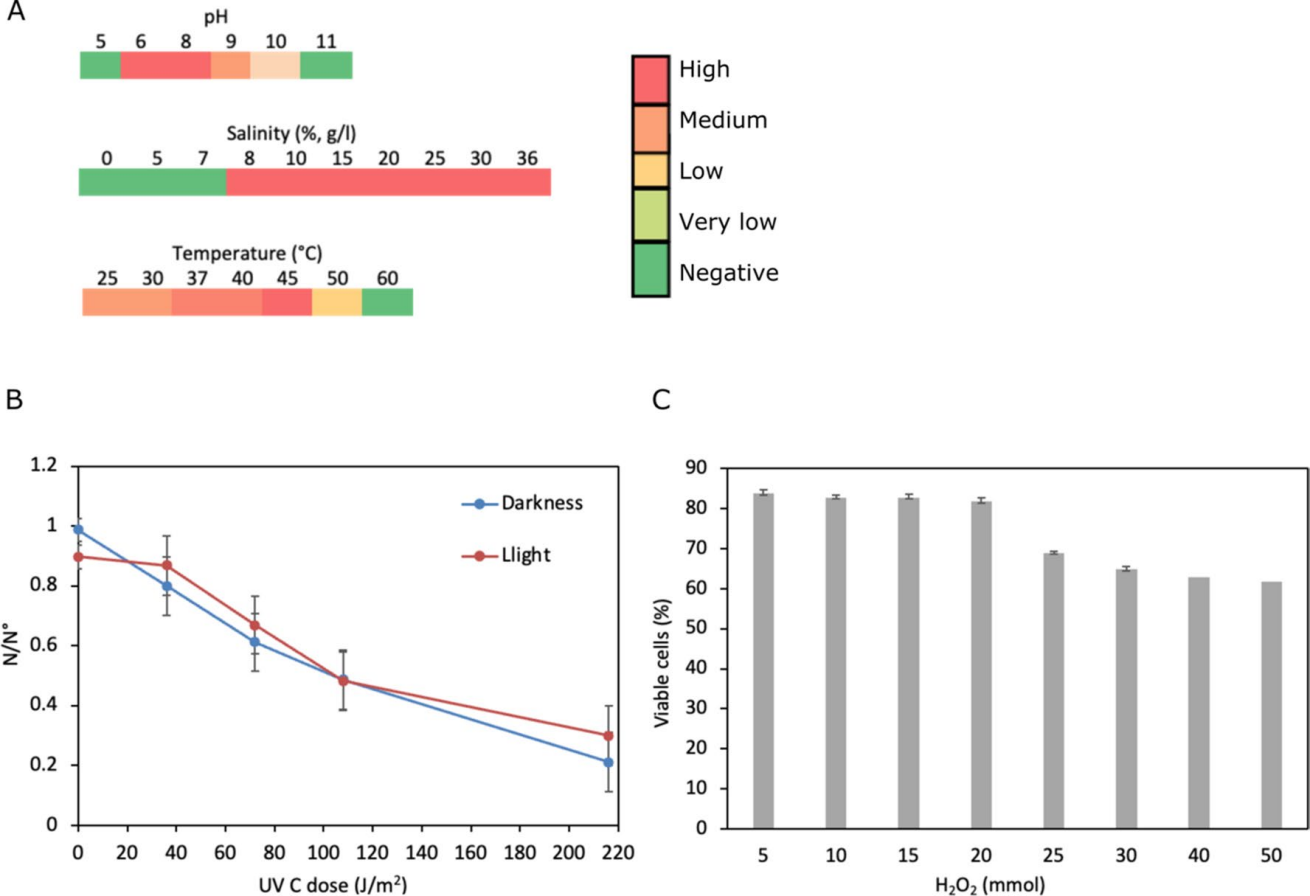
Physiological tolerance of *N. altunense* 4.1R to abiotic stress. **A** Heat map plots of physiological tolerance to pH (at 37 °C), salinity (at 37 °C and pH 7.4), and temperature (at pH 7.4). **B** Survival cells after exposition to increasing UV-C radiation after incubation in light and dark conditions. **C** Percentage of cells viability at different concentrations of H_2_O_2_. The bars represent the standard error, which is not shown at 40 and 50 mM H_2_O_2_ due to their small value

*Natrinema altunense* 4.1R grew in salinities ranging from 8 to 36% (w/v) and in temperatures from 25 to 50 °C but grew optimally at 45 °C. The optimal pH was 7.4, although the strain was able to grow in a range of 6–10 (Fig. 2A).

#### Tolerance to UV-C radiation

To investigate the UV-C radiation resistance of all isolates, cells were exposed to increasing doses of UV-C radiation (UVr-C). The results showed no difference between recovery under light or dark conditions (Fig. 2B), indicating that the repair mechanisms were mainly light-independent. *N*. *altunense* 4.1R showed a UV-C radiation resistance withstanding up to 180 J/m^2^ with no loss of viability and lost 50% of viability only when exposed to radiation with 260 J/m^2^.

#### Hydrogen peroxide-induced oxidative stress response

In order to assess the tolerance to hydrogen peroxide-induced oxidative stress at different concentrations, cells were subjected to varying concentration of H_2_O_2_ (Fig. 2C). The results showed that the viability of cells remains stable at around 60% in presence of H_2_O_2_ concentration up to 50 mM.

### Insights into the genome of *N. altunense* 4.1R

We opted to sequence the genome of strain 4.1R to identify the genetic determinants of abiotic stress tolerance compared to other sequenced *N. altunense* 4.1R. Below are insights gained from analyzing genome sequence.

#### General genomic features

An overview of the genome features of strain 4.1R is shown in Table 1. Genome sequence consists of 81 contigs and 12 Scaffolds with a size of 3,670,000 bp and G + C content of 64.9%. It contains 3487 predicted coding sequences, 2334 (66.9%) of which were protein-coding genes with functional assignments and the remaining 1323 genes were of unknown functions. Besides, a total of 47 tRNA, 4 rRNA loci (5S, 16S, 23S). Functional annotation based on the RAST SEED system showed that 4.1R genes were classified into 607 subsystems with the highest representation of genes (210) involved in amino acids and their derivatives, protein metabolism (116), carbohydrates (111), fatty acids, lipids and isoprenoids (74), Respiration (68), cofactors, vitamins, prosthetic groups, pigments (52), DNA metabolism (54), Cofactors, Vitamins, Prosthetic Groups, Pigments (52), Nucleosides and Nucleotides (43), RNA metabolism (41) and Membrane Transport (32). According to the COG annotation, the CDSs of 4.1R genome were classified into 20 functional categories where the majority (25%) were assigned to unknown function cluster, then to amino acid metabolism and transport (9%) (Fig. S2). The strain is equipped with a variety of motility related genes, i.e., flagella biosynthesis, as well as replication and repair genes potentially involved in DNA damage repair caused by ultraviolet radiation. Compared to other genome sequences of *N. altunense* strains available in NCBI database (*N*. *altunense* AJ2 (JNCS01000019), *N*. *altunense* JCM 12,890 (AOIK00000000) and *N*. *altunense* 1A4-DGR (JXAN00000000.1), this functional profile is similar (Fig. S2).

**Table 1:** Genome properties and features of *N. altunense* 4.1R

Property	Total
*DNA, total number of bases*	3,670,000
DNA coding number of bases	3,085,696
DNA G + C number of bases	2,378,236
*DNA GC*	64.9
*N50/ L50/ DNA scaffolds*	99,213/11/12
*Genes total number*	3664
*Protein coding genes*	3487
RNA genes	53
rRNA genes	4
(5S rRNA/16S rRNA/23S rRNA)	2/1/1
tRNA genes	47
Other RNA genes	2
Protein coding genes with function prediction	2334
Without function prediction	1323
Protein coding genes with enzymes	763
w/o enzymes but with candidate KO based enzymes	35
Protein coding genes connected to KEGG pathways	838
Not connected to KEGG pathways	2819
Protein coding genes connected to KEGG Orthology (KO)	1414
Not connected to KEGG Orthology (KO)	2243
Protein coding genes connected to MetaCyc pathways	673
Not connected to MetaCyc pathways	2984
Protein coding genes with COGs	2007
With KOGs	632
With Pfam	2415
With TIGRfam	759
Chromosomal Cassettes	254
Fused Protein coding genes	81
Protein coding genes coding signal peptides	71
Protein coding genes coding transmembrane proteins	849
*COG clusters*	1169
*KOG clusters*	423

#### Comparative genomic analysis

Like 16S rRNA gene-based phylogenetic analyses (Fig. 1), the results of ANI and isDDH calculation for the 4.1R genome sequence (Table S2) identified *N*. *altunense* AJ2 (JNCS01000019) as its closest relative with 98.03% ANI similarity and 82.2% isDDH. These values are within the cut-offs to define species (95% ANI) (Rodriguez-R & Konstantinidis 2014) and 70% is DDH (Auch et al. 2010; Meier-Kolthoff et al. 2013). These results confirm the placement of the strain as a member of *N*. *altunense* species.

**Fig. 3:**
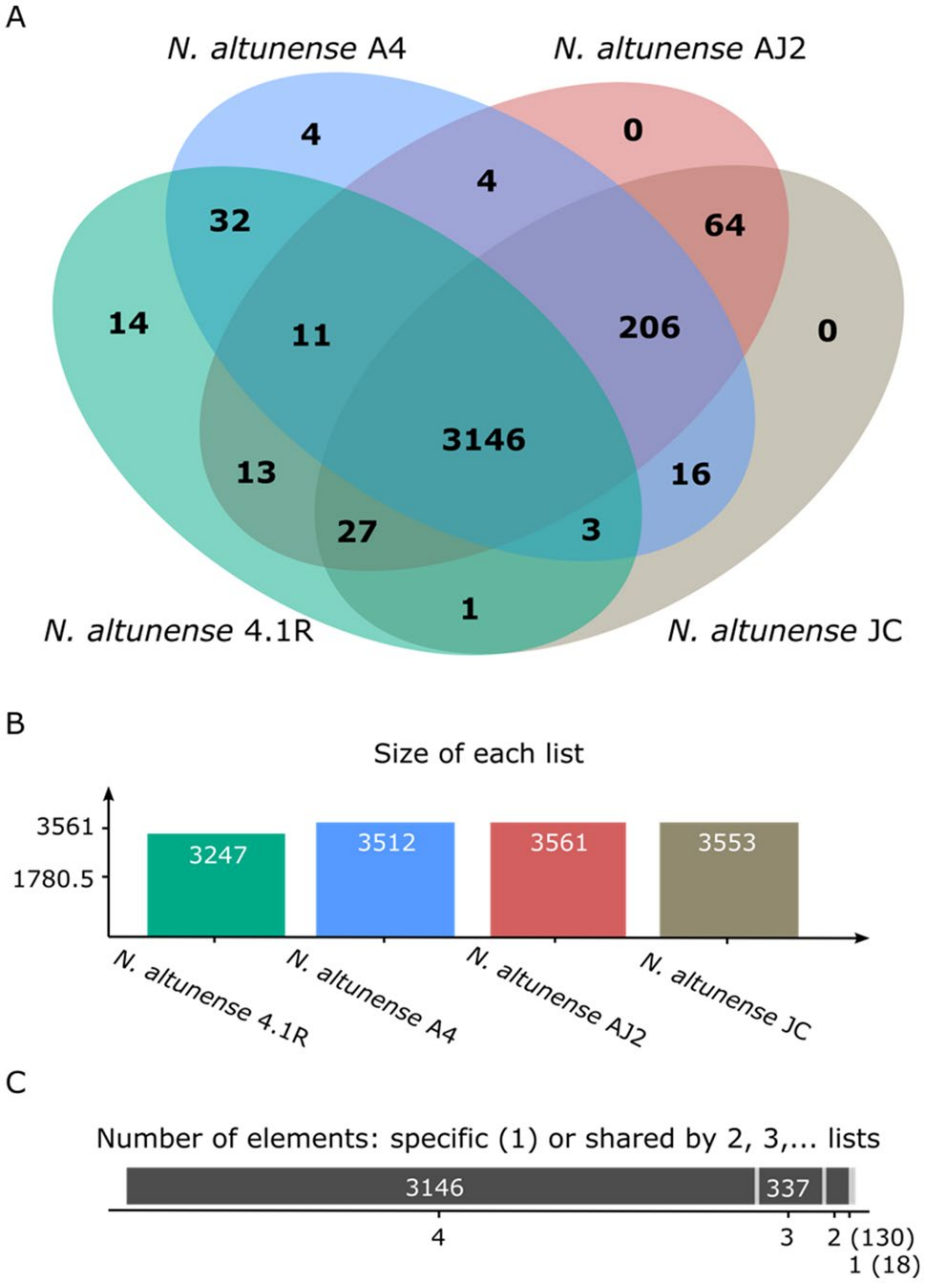
Comparative genome analysis. **A** Venn diagram showing the distribution of shared gene families (orthologous clusters) among *N. altunense* 4.1R, *N. altunense* AJ2, *N. altunense* JCM 12,890 and *N. altunense* 1A4-DGR. **B** Totals of orthologs in each genome that were used to generate the Venn diagram. **C** Sum of the number of genes shared between 4 genomes (total of 3146 genes), between 3 genomes (total of 337 genes), between 2 genomes (total of 130 genes), and a total of 18 singletons specific genes identified for *N. altunense* 4.1R (14 genes) and *N. altunense* 1A4-DGR (4 genes)

OrthoVenn was used to construct a *N. altunense* pangenome using the four genomes. The pangenome consisted of 3631 clusters, with 496 orthologous clusters (clusters with at least contains two copies from one genome) and 3135 single-copy gene clusters indicating few duplication events before speciation (Fig. 3). The *N. altunense* core genome contained 3146 proteins (Fig. 3). Only a few clusters were strain-specific (14 and 4 for strains 4.1R and 1A4-DGR genomes, respectively) potentially coding for orphan genes. Functional analysis of these 18 clusters showed that only one of them (a 4.1R-specific cluster) had a functional prediction (encoding sulfuric ester hydrolase activity) while the other clusters remained unannotated (with no Swiss-Prot Hit or Gene Ontology Annotation) (Table S3). These results showed that despite the high percentage of ANI between the four *N. altunense* strains compared, each genome encodes several unique features, and metabolites biosynthesis capabilities, which may be due to the niche ecological adaptation. Besides, a total of 206 orthologous protein-coding genes were unraveled across all genome sequences, except for the 4.1R strain. Molecular function cluster analysis indicated that the majority of the protein-coding genes (73%) were of unknown functions. The rest (23%) were categorized on the basis of orthologous group annotation (COG) into 3 classes belonging to metabolic processes with uneven distribution (i) class P (67. 3%) responsible for inorganic ion transport metabolism (ii) class F (23.07%) involved in nucleotide transport metabolism (iii) class C (9.6%) involved in energy production and conversion. Globally these clusters lost in strain 4.1R probably evolved additional functions in relation with ecological niche adaptation.

### Genomic insights and genetic determinants of abiotic stress response in *N. altunense* 4.1R

#### Strategies and genes related to Halo-adaptation

**Fig. 4:**
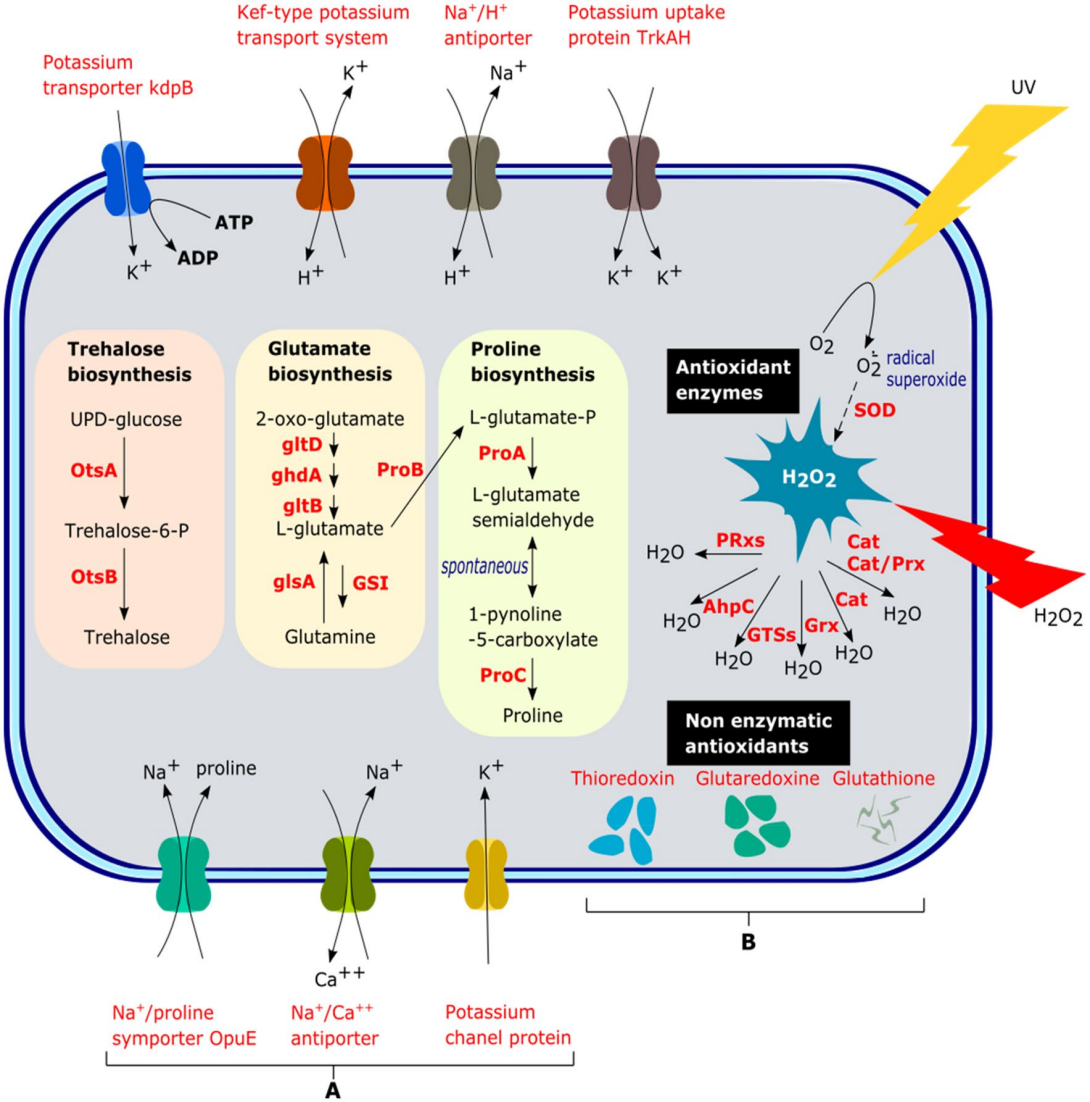
Schematic representation of salt and oxidative stress tolerance mechanisms in *N.* *altunense* 4.1R inferred from whole genome analysis. **A** Genes involved in trehalose, Glutamate, and proline biosynthesis. Various ion transporters such as the potassium transporter (kdpB), Na + /H + antiporter, potassium uptake protein (TrkAH), Na^+^/proline symporters (Opua), potassium channel protein (PCP), sodium/calcium antiporter and Kef-type K^+^ transporter systems were detected to maintain the internal ion homeostasis. **B** Genes involved the oxidative stress tolerance include enzymatic and nonenzymatic antioxidant proteins. Primary enzymatic antioxidants include superoxide dismutase (SOD), catalase (Cat), catalase/peroxidase HPI (Cat/Prx HPI), glutaredoxins (GRx), peroxiredoxins (PRxs), glutathione S-transferases (GST), and alkyl hydroperoxide reductase (AhpC). Primary nonenzymatic antioxidants contain glutathione, thioredoxin, and glutaredoxin

*N. altunense* 4.1R genome encodes several genes potentially involved in osmotic stress response (Table S4, Fig. 4). These include proteins of the potassium uptake trkA, trkH, and KdpB systems and the glutathione-gated potassium efflux Kef (KefA) system known to be responsible for the release of cytoplasmic ions and solutes to reduce the turgor pressure. In addition to potassium accumulation, the genome also encodes several homologs of Na^+^/H^+^ antiporters and monovalent cation/H^+^ antiporters, potentially contributing to the Na^+^/H^+^ homeostasis and Na^+^ tolerance (Fig. 4, Table S4). On the other hand, the genome encodes several pieces of evidence for compatible solute uptake or biosynthesis. These include trehalose biosynthetic machinery from UDP-glucose using trehalose-6-phosphate synthase (otsA) and trehalose-6-phosphatase (otsB) system, and amino acids (for instance, glutamine, glutamate, and proline) biosynthesis (Fig. 4). In addition, genes homologous to compatible solute symporters (for example, Na + /proline (OpuE), proton/glutamate, sodium/glutamate, and Na + /solute) were also identified in the genome and could potentially be employed in the import of these compatible solutes intracellularly during osmotic stress (Fig. 4, Table S4).

#### Genomic basis of UV-C radiation tolerance

*N. altunense* 4.1R genome was queried for mining homologs of genes involved in the above repair mechanisms. Thirty-six genes encoding a complete set of enzymes for nucleotide excision repair (NER) (*n* = 8), mismatch repair (MMR) (*n* = 11), and homologous recombinational repair (HRR) (*n* = 17) were identified (Table S5). These include: (i) UvrABCD excinucleases, UvrD helicase, and DNA ligase (ligA) involved in the NER pathway; (ii) DNA mismatch repair proteins (MutS, MutH, MutL) and RecJ-like exonuclease involved in MMR pathway; (iii) DNA double-strand break repair protein, ATPase and nuclease (Mre11, Rad50, and NurA respectively), Bipolar DNA helicase (HerA), DNA repair and recombination protein (RadA, B), and recombinase (RecA), all involved in HHR pathway. These results highlight the mosaic nature of the DNA repair pathways in *N. altunense* 4.1R.

Putative genes involved in protective mechanisms, such as membrane pigments, including carotenoid C50 and bacteriorubine, have also been identified. In fact, genes encoding carotenoid biosynthesis enzymes, including 15-cis-phytoene synthase (crtB) (locus tag ELS17_RS03615), 1-hydroxy-2-isopentenylcarotenoid 3,4-desaturase (crtD), lycopene beta-cyclase (crtY) (locus tag ELS17_RS02895), phytoene desaturase (crtI) (ELS17_RS15240) and beta-carotene 15,15’-dioxygenase, Brp/Blh family (locus tag, ELS17_RS02900) were found in the genome. Several genes needed for the biosynthesis of bacterioruberin have been identified, including lycopene elongase (locus tag ELS17_RS02895) and bisanhydrobacterioruberin hydratase (cruF, ELS17_RS15230).

#### Genomic basis of oxidative stress tolerance

**Fig. 5:**
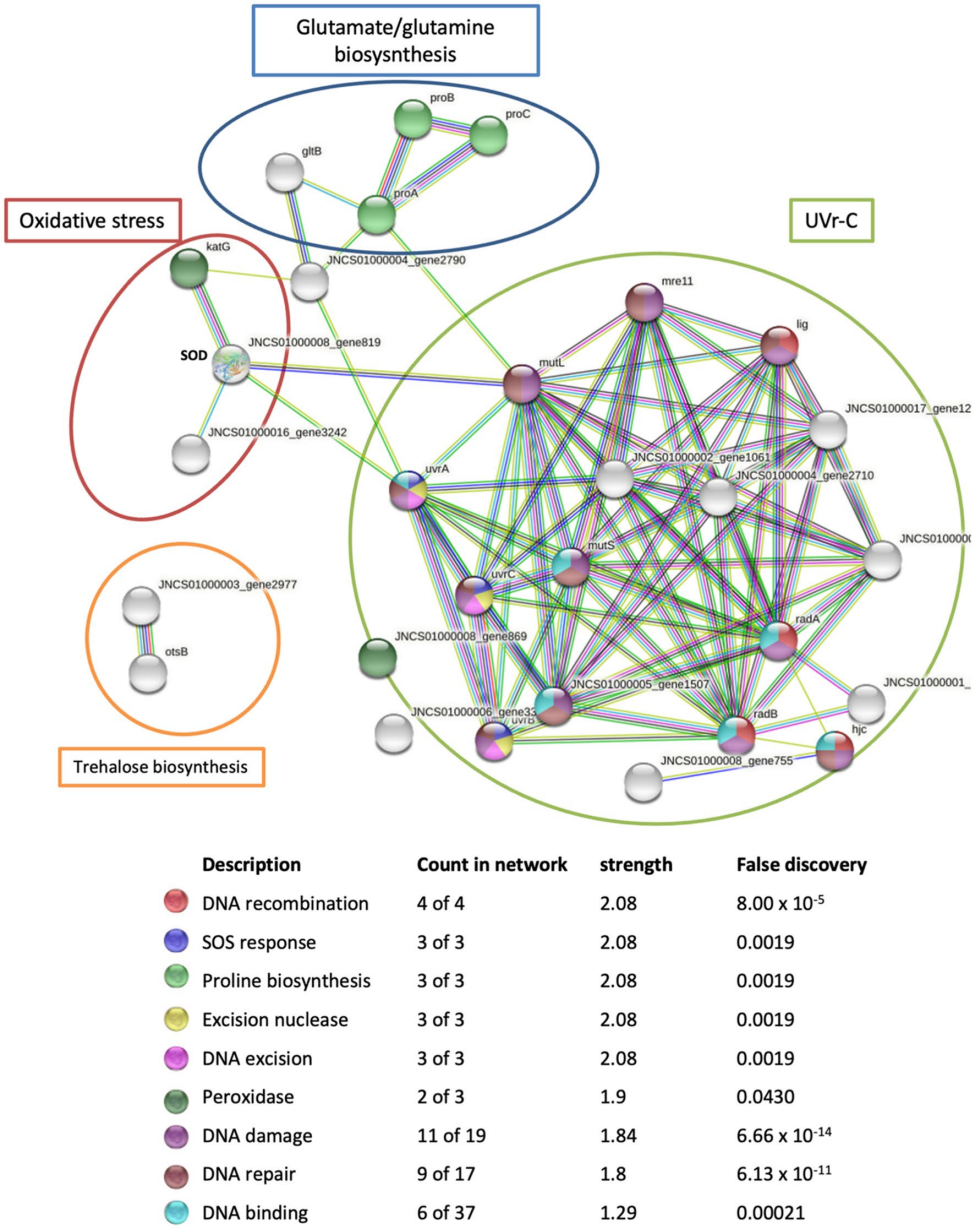
Predicted network interactions of proteins implicated in responses to Uvr-C radiation, osmotic and oxidative stresses of *N. altunense* 4.1R using tools and databases that can predict protein function (PPI enrichment *p*-value: PPI enrichment p-value: < 1.0 $\times$ ^−16^). Colored edges represent the evidence of protein–protein associations where turquoise and pink edges represent known interactions; green, red, and blue edges represent predicted interactions, and the rest represent other interactions like homology prediction. Colored nodes indicate the different metabolic pathways

To cope with oxidative stress, microorganisms use defense mechanisms including antioxidant enzymes as well as nonenzymatic elements. Strain 4.1R genome encodes the enzymatic antioxidants superoxide dismutase (SOD), catalase (Cat), catalase/ Peroxidase HPI (Cat/Prx HPI), glutaredoxins (GRx), peroxiredoxins (PRxs), glutathione-S-transferases (GSTs), alkyl hydroperoxide reductase (AhpC), all of them transform the generated H_2_O_2_ into a neutral element (H_2_O). As well, non-enzymatic antioxidants that are capable of trapping free radicals, e.g., glutathione, thioredoxin and glutaredoxin (Table S6, Fig. 5) were also encoded by 4.1R genome. In addition, gas vesicles were also implicated in oxidative stress defense (Winter et al 2018) by controlling the cell buoyancy and hence oxidative stress avoidance. Several genes were identified in *N. altunense* 4.1R genome including gas vesicle protein GvpN (locus tag, ELS17_RS10380), gas vesicle structural protein (GvpA) (locus tag ELS17_RS10385) and gas vesicle protein GvpFL (locus tag ELS17_RS10420).

### In silico* protein–protein interaction network*

Analysis of protein–protein interactions indicated possible interactions between the different proteins involved in UVr-C, osmotic and oxidative stress tolerance simultaneously (Fig. 5). In fact, the connection between the proteins involved in the UVr-C tolerance and the glutamate/proline biosynthesis involved in osmoadaptation is ensured via the connection between the two proteins mutL and ProA. As regards glutamate/proline biosynthesis system, is connected to the catalase-peroxidase (katG) proteins of the oxidative system via the ammonium transporter protein belonging to the P(II) protein family (JNCS01000004_gene2790). The oxidative system is also linked to the MutL and UVra proteins of the UVr-C system via the SOD proteins.

### Molecular modeling of putative proteins involved in abiotic stress tolerance

The 3D molecular structures of seven proteins were constructed by homology modeling. These proteins are related to responses to UV-C radiation (excinucleases UvrA, UvrB, and UvrC, and photolyase), saline stress (trehalose-6-phosphate synthase (OtsA) and trehalose-phosphatase (OtsB)), and oxidative stress (superoxide dismutase (SOD)) (Table 2). According to the validation, all the models can be considered reliable, the Z-score gave satisfactory or good scores (Table 2). A Z-score, as it is defined in YASARA software, describes how many standard deviations the model quality is away from the average high-resolution X-ray structure. Negative values indicate that the homology model looks worse than a high-resolution X-ray structure. Under this context, the best model corresponds to SOD (Z = −0.063), while the UvrC and OtsA corresponds to the worst (Z = −1.867 and Z = −1.936 respectively). This is due to the lack of crystallographic structures with significant similarities to UvrC an OtsA. Beyond, all proteins, except OtsA, were modeled using their full sequences. OtsA was modeled from the amino acid 29–5530, which is 92.4% of the total sequence. On the other hand, none of the proteins have shown signal peptide, which indicates that all of them are intracellular.

**Table 2:** Modelled proteins

Protein	Modeled region	Oligomerization	ligands	Templates (PDB)	Z-score	Comment
UvrA	1–986	Dimer	Zn	3UWX, 2R6F, 2VF7, 3PIH	−1387	Satisfactory
UvrB	1–286	Monomer	ADP, polythymine trinucleotide	2D7D, 1T5L, 6O8G	−0483	Good
UvrC	1–619	Monomer		2NRT, 5HM5, 3C65, 3C1Y, 1YD6	−1867	Satisfactory
Photolyase	1–469	Monomer	FAD	1OWL, 1DNP, 5ZM0, 2E0I	−0387	Good
OtsA	29–553	Tetramer	UDP, Trehalose-6 phosphate	6JBR	−1936	Satisfactory
OtsB	1–282	Monomer	Mg	5GVX, 5DXL, 5GVX, 6UPB, 1U02	−0748	Good
SOD	1–200	Tetramer	Mn	3EVK, 5VF9, 3AK2	−0063	Good

The PDB codes in bolds are the main template used in the modelling. The Z-score, as it is defined in YASARA software, describes how many standard deviations the model quality is away from the average high-resolution X-ray structure

**Fig. 6:**
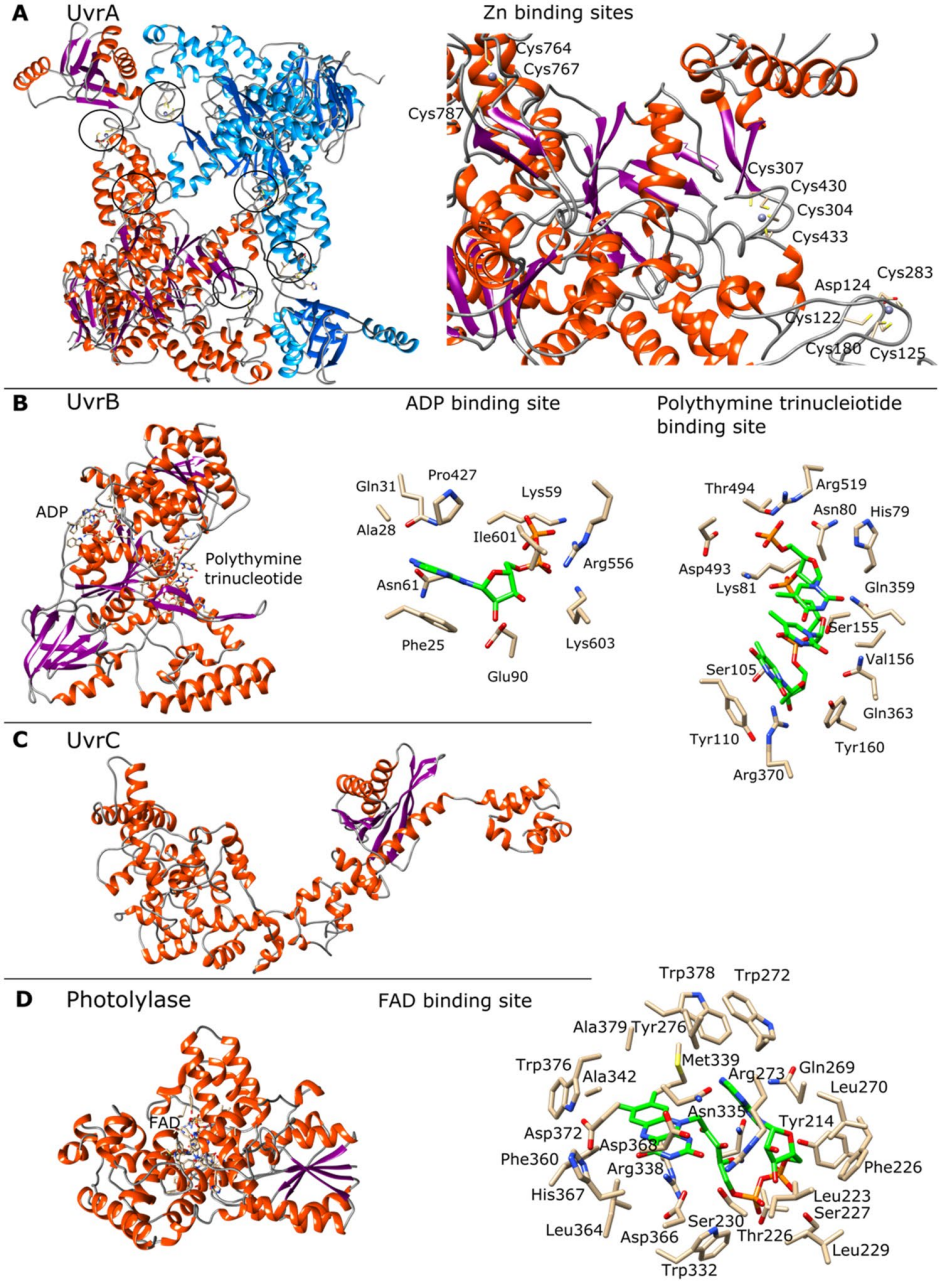
Molecular models of the proteins from *N. altunense* 4.1R involved in responses to UV-C radiation. **A** UvrA. Dimeric structure, where subunits are represented in red and blue. Zn binding sites are indicated in circles and shown in detail right panel. **B** UvrB. Overall structure and details of the ADP and polythyimine trinucleotide binding sites. Ligands are represented with carbon atoms in green, oxygen in red, and nitrogen in blue. **C** UvrC. Overall structure where the alpha helixes are represented in orange, while beta strands in purple. **D** Photolyase. Overall structure and details of the FAD binding site, where the ligand is represented with carbon atoms in green, oxygen in red, and nitrogen in blue

The excinulclease UvrA has been modeled as a dimer (Fig. 6A). The main template was the crystallographic structure of UvrA (PDB code 3UWX) from *Geobacillus stearothermophilus* (Pakotiprapha et al. 2012) which shares 58.39% of identity and a coverage of 97%. The dimeric structure forms a central channel, in which DNA binds. Three zinc fingers were deduced, which are constituted by cysteines (Fig. 6A). The excinuclease UvrB model has been obtained as a monomer, binding ADP and polythymine trinucleotide (Fig. 6B). The main template was the crystallographic structure of UvrB (PDB 2D7D) from *Bacillus subtilis* (Eryilmaz et al. 2006), which shares 57.62% of identity and a coverage of 94%. The amino acids surrounding the adenine moiety are Phe25, Ala28, Gln31, Asn61, and Pro427. Phe25 makes stacking interactions with the purine ring of adenine. The sugar moiety does not show a direct hydrogen bonding interaction with any amino acid of the minding site; however, there is enough room for a water molecule, between the hydroxyl groups of the ribose and Glu90. It is notable that the phosphate groups of ADP interact with Lys603, Arg556 and Lys59. The last amino acid interacts with phosphate β. On the other hand, the polythymine trinucleotide ligand exhibits a conformation where the thymine rings pack together through stacking interactions. The 3′-OH end is surrounded by Tyr110 and Tyr160, which interact with the deoxyribose. The carbonyl 2 from the thymine ring interacts with the Arg370, while the phosphate group interacts with Ser155 and Ser157. The phosphate from the second nucleotide makes salt bridge with Lys81, while the thymine ring is stabilized by Gln360, and the deoxyribose by the Val156. The 5′-phosphate end binds to Arg519. Deoxyribose appears to be stabilized by a hydrogen bond between the oxygen of furanose and a donor group of the imidazole residue of histidine 79. Finally, the thymine ring interacts with the Gln359 (Fig. 6B).

The UvrC excinuclease model obtained is a ligand-free monomer (Fig. 6C). The main template used was the UrvC crystallographic structure (PDB 2NRT) from *Thermotoga maritima* (Karakas et al. 2007), which shares 44.28% identity and 31% coverage. Due to the low coverage, it was difficult to build a good model, although the validation of the hybrid model gave a satisfactory precision (Table 2). This protein is an interesting candidate for study by X-ray crystallography or other experimental technique, as no significant homologues were found in the protein data bank.

The model of photolyase was obtained as a monomer complexed with flavin adenine dinucleotide (FAD) (Fig. 6D). The main template was a crystallographic structure of the photolyase (PDB 1OWL) from *Synechococcus elongatus* (Kort et al. 2004). Both proteins share 39.04% of identity with a coverage of 99%. The flavin group is surrounded by the Ser230, Arg338, Met339, Ala347, Phe360, Asp366, Asn371, Trp376, and Ala379. The adenine ring is surrounded by the Gln269, Trp272, Asn335 and Trp378. Despite of several aromatic amino acids are present in the FAD binding site, no stacking interactions between the enzyme and FAD were observed. Phosphate groups are surrounded by Tyr 214, Thr226, Ser227 and Trp332 while the ribose moiety does it by Leu233, Leu270, and Arg273. All these amino acids are conserved in the template used.

**Fig. 7:**
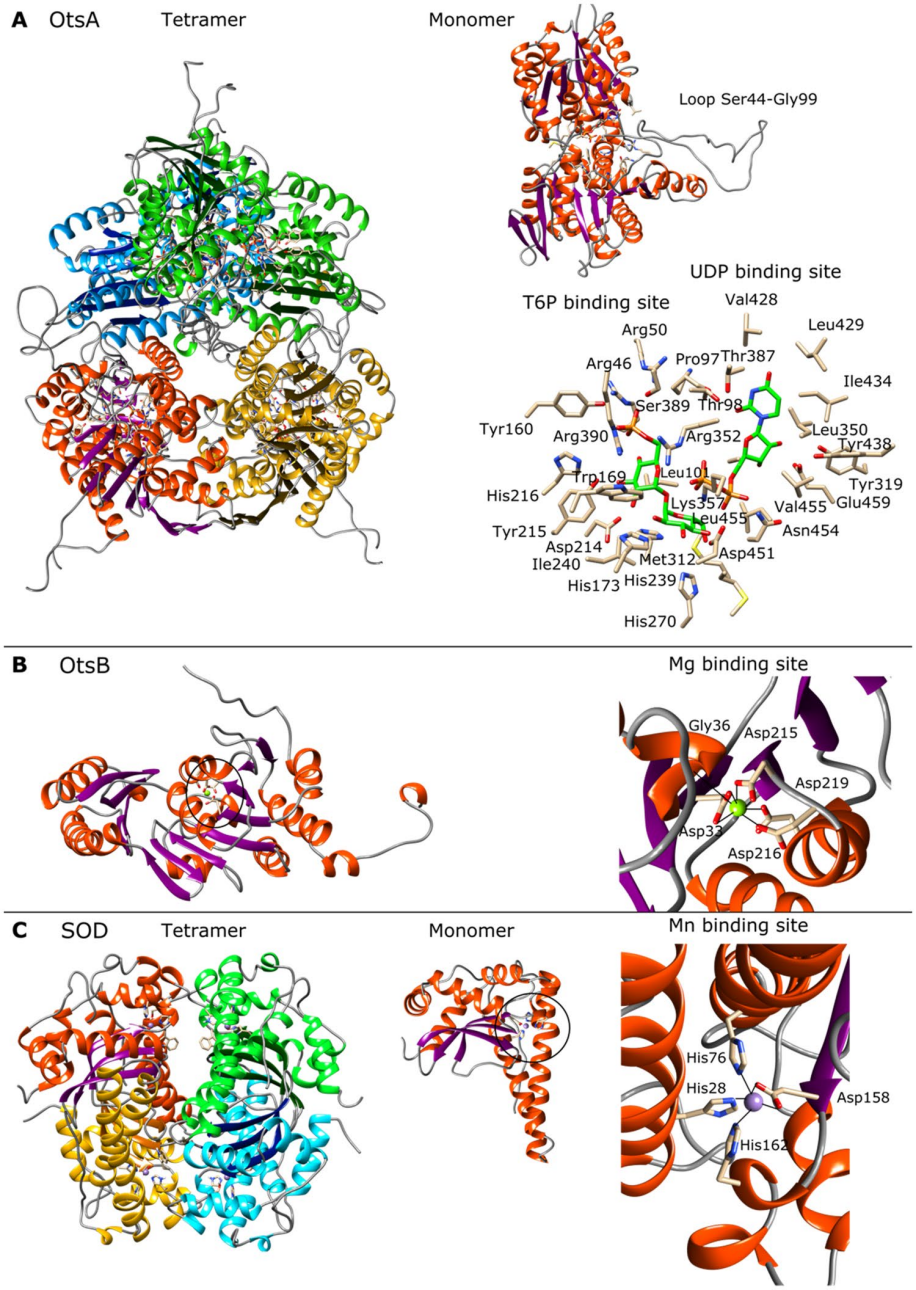
Molecular models of the proteins from *N. altunense* 4.1R involved in responses to saline (OtsA and OtsB) and oxidative stress (SOD). **A** OtsA. Tetrameric structure, subunit, and details of the T6P and UDP binding sites. In the tetramer, each subunit is represented by a different color. In the right-top a subunit is shown where a flexible loop (Ser44-Gly99) is remarked. In the right-down, the T6P and UDP are represented with carbon atoms in green, oxygen in red, and nitrogen in blue. **B** OtsB. Overall structure and details of the Mg binding site. Notice the conserved aspartates coordinating the Mg binding. **C** SOD. Tetramer, details of the subunit and Mn binding site. Each subunit is represented by a different color in the tetramer. Notice that the Mn binding site is coordinated by three conserved histidines and one aspartate

The trehalose-6-phosphate synthase (OtsA) model was obtained as a tetramer and as a complex with uridine diphosphate (UDP) and trehaolose-6-phosphate (T6P) (Fig. 7A). The template used was the trehalose-6-phosphate synthase (PDB 6JBR) from *Pyricularia oryzae* (Wang et al. 2019). The identity and coverage of both proteins were 32.66 and 77% respectively. Although, this is the less accurate model according to the Z-score, the amino acids involved in the ligands binding sites are well conserved. This enzyme is a glycosyltransferase family 20 (GT20). The monomeric subunit has a typical GT-B two-domain fold. The catalytic site is in the interdomain region (Fig. 7A). The C-terminal domain has a long loop, from Ser44 to Gly99, that extends towards the inter-domain region, and it is exposed to the solvent. This loop adopts different conformation in each subunit, which suggest it is flexible. T6P phosphate binds to Arg46, Ar50, Arg390, and Tyr360. The reducing end glucose could form hydrogen bonds with the His200, His270 and Asp451, while the non-reducing end glucose, could do it with the Asp241 and Tyr215. The UDP phosphate is binding to Arg352, and Lys 357, while the α, do it with Asn454. The ribose moiety makes hydrogen bonds with the Glu459. The uracil ring is surrounded by Leu350, Thr387, Ile434, Leu429, Val 420, and Thr98. The last make a hydrogen bond with the 2-ketonic group of the uracil.

The trehalose-phosphatase (OtsB) model was obtained as a monomer binding a magnesium metal ion (Fig. 7B). The main template was a crystallographic structure of the trehalose-phosphate (PDB 5GVX) from *Mycobacterium tuberculosis* (Shan et al. 2016). Both proteins share 34.2% of identity with a coverage of 85%. A Mg binding site was deduced, which includes Asp22, Asp 215, Asp216, Asp219 and the backbone of Gly36 (Fig. 7B).

The superoxide dismutase (SOD) model obtained is a tetrameric structure with a magnesium binding site in each subunit (Fig. 7C). The main template used was the crystallographic structure of the superoxide dismutase (PDB 3EVK) from *Pyrobaculum aerophilum* (Lee 2008). Both proteins share 43.43% of identity with a coverage of 97%. The Mn binding site is formed by His28, His76, His162 and Asp158 (Fig. 7C).

## Discussion

Haloarchaea constitutes one of the most predominant groups of the microbial community present in hypersaline habitats (Baati et al. 2022; Martínez et al. 2022; Najjari et al. 2015; Najjari et al. 2021). Adaptations in such harsh environments render the genome of halophilic archaea abundant in many essential genes that are lacking in other microorganisms (Feng et al., 2021). The use of genome-based approaches for certain halophilic archaea paves the way for a molecular understanding of this adaptation (Gaba et al. 2020; Pfeiffer and Oesterhelt 2015). The main objective of this study was to explore the physiological and genomic determinants of the *N. altunense* 4.1R strain isolated from Sabkhat Ennaouel that enable tolerance to abiotic stresses, including osmotic, UV-C radiation, and oxidative (H_2_O_2_).

Isolation of *N. altunense* 4.1 R strain was performed on DSC-97 medium containing 20% of NaCl (Najjari et al. 2021), colonies were red, and cells were rod-shaped. Phenotypic properties of the colonies closely resembled those published for haloarchaea isolated from hypersaline habitats (Najjari et al. 2021; Xu et al. 2005). In fact, several studies have reported that most members of the family Halobacteriaceae are known to produce red, orange, or pink colonies on agar plates due to the production of carotenoids in their cell membranes or with the C50 compound bacterioruberin and its derivatives (Squillaci et al. 2017). These pigments are known to protect haloarchal cells from abiotic stress and are also involved in maintaining membrane fluidity and the photoresist system (DasSarma et al. 2020a, b).

Based on 16S rRNA gene sequencing 4.1R strain was assigned to *N*. *altunense* AJ2 (NR_112855.1) with 99.68% of similarity. AJ2 was isolated from a high-altitude salt lake in Xinjiang, China (Kaur et al. 2010). The whole genome sequence analysis confirms the affiliation of strain 4.1R to the species *N. altunense* based on ANI (98.03%) and isDDH (82.2%) similarities values with the genome sequence of *N. altunense* AJ2 (NZ_JNCS00000000.1), these values are within the cut-offs used to define species (95% ANI) (Rodriguez-R & Konstantinidis 2014) and 70% isDDH (Auch et al. 2010; Meier-Kolthoff et al. 2013).

*N. altunense* 4.1R showed an ability to grow at salinities from 8 to 30% (w/v), with optimal growth at concentrations ranging from 20 to 30% (w/v). Haloarchaea are obligate halophiles that need a minimum of 8% w/v NaCl to grow (Oren 2008), Oren et al. (2009), and they can survive up to saturated salt (> 30% w/v) (Najjari et al. 2021; Youssef et al. 2014). Various haloarchaea-related 16S rRNA gene sequences were obtained from low salt concentrations samples, like sediments and saline water, by culture-independent methods (Elshahed et al. 2004; Najjari et al. 2015; Purdy et al. 2004). *Natrinema* genus members were identified in samples from similar Tunisian Sabkhat ecosystems with salinities ranging from 3 to 37% (Najjari et al. 2021).

Generally, haloarchaea deploys two main strategies to regulate its internal and external osmotic pressure:

(i) The first salt-in strategy uses inorganic ions to balance the internal and external osmotic pressure by transporting sodium ions through Na^+^/H^+^-antiporter, the uptake of K^+^ via trk systems (Trk, Kdp, Ktr, and Kup) and the potassium efflux using kef system (Aston et al., 2007; Becker et al. 2014; Gunde-Cimerman et al. 2018). Those systems were identified in the *N. altunense* 4.1R genome sequence. Indeed, the glutathione-gated potassium efflux Kef (KefA) is known to be responsible for the release of cytoplasmic ions (Booth & Louis 1999; Kung et al. 2010). Most transporters are found in almost all Haloarchaeal strains. However, the TP-driven K^+^ transport system (KdpB) was absent in *Natrinema* sp. J7-2 strain. Indeed, a large number of genes homologous to Na^+^/H^+^ antiporters, and monovalent cation/H^+^ antiporters were identified (Slonczewski et al. 2009). In fact, within haloarchaea, the K + system is considered the primary osmoprotectant strategy rather than compatible solutes in the high-salt environment due to its stability and the high transport efficiency in the cell membrane (Edbeib et al. 2016; Oren 2008; Oren et al. 1999; Youssef et al. 2014; Feng et al. 2012).
(ii) The second, the salt-out strategy, entails the biosynthesis and/or uptake of organic compatible solutes at low concentrations in the cytoplasmic medium to increase internal osmolarity without increasing cytoplasmic salinity (Roberts 2005). Compatible solutes include sugars (trehalose) and polyols, amino acids (glutamine, glutamate, proline), and derivatives (betaines, ectoines) (Edbeib et al. 2016; Gunde-Cimerman et al. 2018; Noha et al. 2014; Roberts 2005). This strategy is considered more flexible than the salt-in strategy in terms of osmoregulation, and the microorganisms able to utilize compatible solutes can tolerate a broader range of salinity (Youssef et al. 2014). Here, the genomic analysis suggests that the *N. altinense* 4.1R synthesizes trehalose using the OtsAB pathway like reported in other haloarchaea such as *Haladaptatus paucihalophilus* (Youssef et al. 2014) and *Halococcus hamelinensis* (Gudhka et al. 2015). These species are reported to tolerate low salt concentrations using the compatible solute strategy. Trehalose is a non-reducing disaccharide used in low salinity concentrations (Najjari et al. 2015; Youssef et al. 2014). It acts as an osmoregulatory agent and provides protection against high- and low-temperature stresses (Desmarais et al., 1997). Trehalose can be biosynthesized by the OstAB, TreS, TreP, TreY, and TreT pathways (Avonce et al. 2006). Previous studies have also reported that some halophilic cells accumulate glutamate and glutamine in high concentrations, compatible with osmolytes (Falb et al. 2008; Saum & Müller 2007; Shiyan et al. 2014). The glutamate could be converted to proline by an alternative pathway in some haloarchaea strains. In the case of the halophilic archaea *H. hamelinensis, *salinity levels, and glutamate concentration have been reported to trigger the change of osmotic strategies during the transition from moderate to high salinity. In halophiles generally, the uptake of compatible solutes is performed by multiple porters, such as the symporters and ABC transporters (Falb et al. 2008; Saum & Müller 2007; Shiyan et al. 2014). Here, we found some symporters like Na^+^/proline (OpuE), proton/glutamate, sodium/glutamate, and Na^+^/solute (Becker et al. 2014; Edbeib et al. 2016; Jung et al., 2012).

Halophiles can also tolerate high UV radiation, which causes DNA damage and lesions. To avoid these negative effects haloarchaea employ several tolerance mechanisms such as nucleotide excision repair (NER), mismatch repair (MMR), and homologous recombination repair (HRR) (Baliga et al. 2004; DasSarma et al., 2001; Leuko et al. 2011). Here, we assessed the tolerance of N. altinense 4.1R strain to UVr-C radiation. The results showed a survival rate near 100% up to 180 J/m^2^ with no difference between recovery under light or dark conditions, indicating that the repair mechanisms were mainly light-independent. The same profile has been observed within *H. salinarum* NRC-1, exhibiting a survival rate close to 100% up to 110 J/m^2^ (Crowley et al. 2006). *H. salinarum* is reported to be an UVr-C-resistant model able to survive at high UVr-C rates, among others, due to its effective mechanisms to recognize and repair DNA damage after UVR exposure (Baliga et al. 2004).

Mining the *N. altunense* 4.1R genome sequence revealed the expected homologous proteins and pathways involved in UVr-C tolerance, including NER, MMR, and HRR systems. The genes of the NER system comprise UvrAB (for damage recognition), Uvr-C (acting as exinucleases), UvrD (a DNA helicase II), and DNA ligase (ligA). These genes were previously identified in certain halophiles, such as *Halobacterium cutirubrum*, *Halobacterium halobium*, *H*. *volcanii*, and *H*. *salinarum* (Baliga et al. 2004; Crowley et al. 2006; McCready et al. 2005). NER is a universal system controlling the stability of the chromosomes in the cells (Capes et al. 2011; Hoeijmakers 2001; Kish & DiRuggiero 2012; Morita et al. 2010; Sancar 1996; Zhao et al. 2006). Earlier studies have shown its involvement in the damage restoration induced by Uvr-C in *H*. *hamelinensis*, *H*. *volcanii*, and *H*. *salinarum* NRC1 (Baliga et al. 2004; Crowley et al. 2006; Lestini et al. 2010; Leuko et al. 2011). Moreover, MutL/MutS/MutH and RecJ-like exonucleases genes implicated in MMR systems have been identified. Although these systems have been found in several bacteria and eukaryotes, they are absent in many archaea, except for some halophilic and methanogens ones. The latter would have acquired the genes encoding NER a from bacteria by lateral transfer (Harfe & Jinks-Robertson 2000; Marshall et al. 2007). Finally, the HRR system includes DNA double-strand break repair protein, nucleases (Mre11, Rad50, and NurA), bipolar DNA helicase (HerA), DNA repair and recombination protein (RadA, B), and recombinase. It should be noted that some enzymes from halophiles are homologous to those in eukaryotes, such as the case of RadA from Halobacterium sp. NRC-1, which shows the same function as the eukaryotic RecA/Rad51 proteins (Seitz et al. 1998).

Carotenoids play a key role in the processes of photoprotection (Grivard et al. 2022) and are found to be important in protecting against UV-C radiation in *H*. *salinarium* by scavenging for hydroxyl radicals (Kottemann et al. 2005; Shahmohammadi et al. 1998). It was found that the N. altunense 4.1R genome encodes carotenoid biosynthesis enzymes, including phytoene synthase (crtB) isopentenylcarotenoid (crtD), lycopene beta-cyclase (crtY), phytoene desaturase (crtI) and beta-carotene family in addition to genes involved in bacterioruberin biosynthesis like lycopene elongase and bisanhydrobacterioruberin hydratase like reported in several other haloarchaea (Kottemann et al. 2005; Shahmohammadi et al. 1998).

Exposure to extreme environmental pressures such as UV radiation, hyper salinity, and elevated temperature can generate high levels of oxidative stress species (ROS) such as superoxide (·O2 −), hydrogen peroxide (H_2_O_2_), and hydroxyl radicals (·OH) which affect the different cellular components including proteins, nucleic acid, cell wall components and membrane lipids (Whitehead et al. 2012; Yusuf et al. 2019). Here, we evaluated the oxidative stress response of *N. altunense* 4.1R to the presence of various concentrations of H2O2. Results indicated a high oxidative stress tolerance (60% viability up to 50 mM) compared to *H. mediterranei* and *H. salinarum* NRC1, which tolerated up to 25 mM and 30 mM H2O2, respectively (Kaur et al. 2010). In fact, several defense mechanisms are implicated in oxidative stress, including antioxidant enzymes such as catalase (CAT), superoxide dismutase (SOD), peroxidase, peroxiredoxins; nonenzymatic elements such as glutathione (GSH), thioredoxins, glutaredoxin and glutathione peroxidase (Alscher et al. 2002; Anderson 1998; Carmel-Harel & Storz 2000; Sattin et al. 2015; Shin et al. 2011). Those genes and proteins were found in the *N. altunense* 4.1R genome sequence. Several studies have investigated pathways of ROS detoxification within eukaryotes and bacteria (Gelsinger et al. 2021; Lyall et al. 2020). However, fewer studies have been investigated in haloarchaeal strains. It’s worth noting that two species, *Halobacterium* sp. NRC-1 and *H*. *volcanii* were the most studied strains (Gelsinger et al. 2021; Sharma et al. 2012). On the other hand, gas vesicles were reported to be implicated in ROS defense, especially under low oxygen conditions, which enable cells to float to the surface where light and oxygen concentrations are optimal for growth (Winter et al. 2018; Pfeiffer and Oesterhelt 2015). Here, in the *N. altunense* 4.1R genome, several genes associated with gas vesicle protein (GvpN), gas vesicle structural protein (GvpA), and gas vesicle protein (GvpFL), as well as genes responsible for the construction of the gas vesicle wall and as regulatory proteins, were found. Carotenoids are also widely known for their remarkable antioxidant properties because they can quench free radicals (Shahmohammadi et al. 1998).

Molecular models of key enzymes (Table 2) involved in the abiotic stress response showed highly conserved amino acids at active sites, even with non-closely related species. These results and the Z-scores support the reliability of the modeled structures. Besides, several structures, such as UrvB, photolyase, and OtsA, were obtained with ligands, which allowed the identification of amino acids that stabilize the substrates, providing insights into the structure and function of these enzymes. The models also predicted the presence of metal ion binding sites in UvrA (Zn), OtsB (Mn), and SOD (Mn). Finally, some models revealed the potential oligomeric forms of some enzymes, such as UvrA, OtsA, and SOD, which could have relevance in the function. Thus, the enzyme 3D models strongly support the predicted function of the annotated genes from *N. altunense.*

Taking together the experimental evidence, genomic analysis, and molecular modeling, this research has contributed to a better understanding of the tolerance to several abiotic stress conditions. The genomic landscapes provide insights into the genes involved in abiotic stress, mainly in cross-stress adaptability, in which an adaptive response to one stress can provide acquired resistance to a second phenomenon. A case in point is the enzymes catalase and superoxide dismutase, both of which are involved in responses to osmotic, oxidative, and UV radiation stresses (Matarredona et al. 2020). Furthermore, salt stress also induces the expression of genes related to membrane transporters, osmoprotective solutes, and oxidative stress proteins, in the case of Halolamina sp. YKT1 (Kurt-Kızıldoğan et al. 2017). The carotenoid (C50) is likewise reported to be implicated in oxidative DNA damage induced by UV light, hydrogen peroxide, and osmotic stresses (Alvares and Furtado 2021; Jones and Baxter 2017; Shahmohammadi et al. 1998). Cross-stress protection has been reported in the eukaryotic and bacterial domains (Hackley and Schmid 2019). Here, in silico analysis of putative proteins interactions networks involved in the cross-stress responses, using the STRING database, revealed potential links between UVr-C and osmotic tolerance systems through mutL and ProA proteins. An additional connection between oxidative stress and UVr-C systems is provided by the MutL, Uvra, Uvr-C, and SOD proteins. Similar interactions have already been reported in two halophilic archaea, *H. salinarum* and *H. volcanii*, where the genes coding for ROS proteins and UV-DNA repair genes were simultaneously regulated upon exposure to oxidative stress (Gelsinger et al. 2021; Kaur et al. 2010). An additional study reported the expression MutS and mismatched DNA repair proteins after long exposure to salt stress within the halophilic archaeon *Methanohalophilus portucalensi* (Shih and Lai 2010). In fact, a limited number of previous studies have reported an interaction between molecules assessing the response in members of haloarchaea to at least two concurrent stress conditions.

## Conclusions

In this study, we assessed the physiological response of *N.* *altunense* 4.1R strain isolated from saline water sampled from Tunisian Sabkhat to abiotic stresses. The findings revealed its capacity to withstand a varied range of salt concentrations, high UV-C radiation, as well as oxidative stress. The whole genome analysis revealed characteristic genes associated with its capacity to survive in a polyextremophilic environment. It possesses genes related to osmoadaptation and pH homeostasis, genes involved in different pathways of DNA repair mechanisms, and genes related to the tolerance to oxidative stress. The analysis of 3D molecular structures of modeled proteins related to responses to UV-C radiation (UvrA, UvrB, and UvrC, and photolyase), saline stress (OtsA and OtsB), and oxidative stress (SOD) have shown highly conserved regions, mainly in the catalytic sites and cofactor binding sites, which strongly support their predicted functions. It is also remarkable the computational prediction of oligomeric forms such as UvrA, OtsA, and SOD. This study gives insights to gain, in the future, an in-depth understanding of the regulation of metabolic pathways under stressful conditions.

## Supplementary Information

Below is the link to the electronic supplementary material.

Supplementary file1 (PDF 1043 KB)

## Data Availability

Whole Genome Shotgun project has been deposited at DDBJ/ENA/GenBank under the accession SHMR00000000. The version described in this paper is version SHMR01000000. Sequence for the 16S rRNA gene was deposited in GenBank under the accession number MW534742.1.
