# Supplementary material for: Physiological and genomic insights into abiotic stress of halophilic archaeon Natrinema altunense 4.1R isolated from a saline ecosystem of Tunisian desert
Source: Genetica. 2023 Feb 16;151(2):133–52. doi: 10.1007/s10709-023-00182-0 (PMC9995536; doi:10.1007/s10709-023-00182-0)
Supplement: Supplementary file 1 — Supplementary file1 (PDF 1043 KB) [file 10709_2023_182_MOESM1_ESM.pdf]

Supplementary Materials

Physiological and genomic insights into abiotic stress of halophilic archaeon *Natrinema altunense* 4.1R isolated from a saline ecosystem of Tunisian desert

Afef Najjari[1], Ayoub Boussetta[1], Noha Youssef[2], Javier A. Linares-Pastén[3,*], Mouna Mahjoubi[4], Rahma Belloum[1], Haitham Sghaier[5], Ameur Cherif[4] and Hadda Imene Ouzari[1]

[1] Université Tunis El Manar, Faculté des Sciences de Tunis, LR03ES03 Laboratoire de Microbiologie et Biomolécules Actives, 2092, Tunis, Tunisie
[2] Department of Microbiology and Molecular Genetics, Oklahoma State University, Stillwater, OK, USA
[3] Biotechnology, Faculty of Engineering, Lunds Tekniska Högskola (LTH), Lund University. P.O. Box 124, 22100 Lund, Sweden
[4] Univ. Manouba, ISBST, LR11-ES31 BVBGR, Biotechpole Sidi Thabet, 2020, Ariana, Tunisia.
[5] Laboratory "Energy and Matter for Development of Nuclear Sciences" (LR16CNSTN02), National Center for Nuclear Sciences and Technology (CNSTN)
* Correspondence: javier.linares_pasten@biotek.lu.se

A B

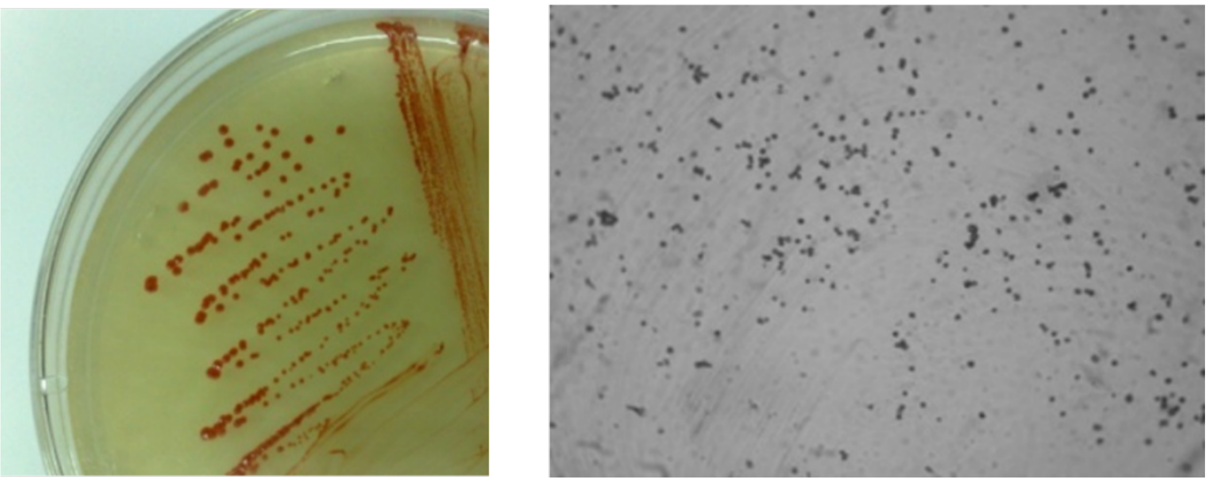

Figure S1. (A) 4.1R colonies have a red color on DSM-97 medium containing 25% of NaCl (B): The cells of strain 4.1R showing rod-shaped such as round visible under microscope at ×100 magnification.

Table S1: Physicochemical analysis of saline water sampled from Sabkhat and Nawal

pH	7.48
Salinity (mg/L)	29500
Conductivity (mS/cm)	109.7
Dry residue (mg/L)	68831
Ca^{2+} (meq/L)	300
Ca^{2+} (mg/L)	6000
Mg^{2+} (meq/L)	220
Mg^{2+} (mg/L)	2640
Na^{+} (meq/L)	553.0
Na^{+} (mg/L)	12719
K^{+} (meq/L)	12
K^{+} (mg/L)	468
Cations (meq/L)	1084.3
$SO_4{}^{2-}$(meq/L)	520
SO_4^{2-} (mg/L)	24960
Cl^{-} (meq/L)	600
Cl^{-} (mg/L)	21300
$NO_3{}^{-}$ (meq/L)	0.03
NO_3^{-} (mg/L)	1.83
HCO_3^{-} (meq/L)	12
HCO_3^{-} (mg/L)	744
Anions (meq/L)	1132

Table S2. Results of ANI and isDDH distances of *N. altunense* strain 4.1R genome versus three other *N. altunense* sequenced genomes.

**Species**	**Strain**	**Accession number (NCBI)**	**ANI value (%)**	**isDDH estimate (%)**
N. altunense	AJ2	JNCS00000000	98.04	82.2
N. altunense	JCM 12890	AOIK00000000	97.93	82.10
N. altunense	1A4-DGR	JXAN00000000.1	97.92	81.1

Table S3. Specific functional clusters of *Natrinema altunense* strain 4.1R

**ID**	**Protein Count**	**Swiss-Prot Hit**	**GO Annotation**
cluster3461	3	N/A	N/A
cluster3462	3	N/A	N/A
cluster3463	3	Q148F3	GO:0008484; F:sulfuric ester hydrolase activity; IEA:InterPro
cluster3543	2	N/A	N/A
cluster3544	2	N/A	N/A
cluster3545	2	N/A	N/A
cluster3546	2	N/A	N/A
cluster3547	2	N/A	N/A
cluster3548	2	N/A	N/A
cluster3549	2	N/A	N/A
cluster3550	2	N/A	N/A
cluster3551	2	N/A	N/A
cluster3552	2	N/A	N/A
cluster3553	2	N/A	N/A

N/A: not affiliated

Table S4. Putative proteins implicated in osmotic stress adaptation inferred from genome sequence of *N. altunense* strain 4.1R (SHMR00000000.1)

	**Locus tag**	**Protein product**	**Protein Name**
Internal ion homeostasis	ELS17_RS03360	WP_130169531.1	TrkA family potassium uptake protein
	ELS17_RS04340	WP_130169672.1	TrkA family potassium uptake protein
	ELS17_RS04345	WP_130170456.1	TrkH family potassium uptake protein
	ELS17_RS13980	WP_130171173.1	TrkA family potassium uptake protein
	ELS17_RS16445	WP_130171552.1	TrkH family potassium uptake protein
	ELS17_RS16450	WP_130171553.1	TrkA family potassium uptake protein
	ELS17_RS16460	WP_130171555.1	TrkH family potassium uptake protein
	ELS17_RS17065	WP_130171642.1	TrkA family potassium uptake protein
	ELS17_RS03490	WP_007108124.1	phosphate uptake regulator PhoU
	ELS17_RS01075	WP_130169182.1	potassium transporter
	ELS17_RS06685	WP_130170026.1	potassium channel protein
	ELS17_RS06735	WP_130170035.1	potassium transporter
	ELS17_RS07930	WP_130170201.1	potassium transporter TrkA
	ELS17_RS07935	WP_130170202.1	potassium transporter TrkA
	ELS17_RS16435	WP_130171550.1	Trk system potassium transporter TrkA
	ELS17_RS08960	WP_130170342.1	two pore domain potassium channel family protein
	ELS17_RS17915	WP_130171768.1	Trk system potassium transporter TrkA
	fig|1227494.5.peg.2852	fig|1227494.5.peg.28 52	Potassium efflux system KefA protein
	fig|1227494.5.peg.549	fig|1227494.5.peg.54 9	Potassium-transporting ATPase B chain
Osmotic stress	ELS17_RS14085	WP_130171188.1	monovalent cation/H+ antiporter subunit D
	ELS17_RS14095	WP_007109264.1	monovalent cation/H+ antiporter subunit B
	ELS17_RS14110	WP_007109261.1	monovalent cation/H+ antiporter subunit F
	ELS17_RS14115	WP_049900357.1	monovalent cation/H+ antiporter subunit E
	ELS17_RS08795	WP_007109628.1	monovalent cation/H(+) antiporter subunit G
	ELS17_RS08820	WP_007109623.1	Na+/H+ antiporter MnhB subunit-related protein
	ELS17_RS09940	WP_130170559.1	Na+/H+ antiporter NhaC family protein
	ELS17_RS10580	WP_130170661.1	Na+/H+ antiporter NhaC family protein
	ELS17_RS13855	WP_049900363.1	Na+/H+ antiporter NhaC
	ELS17_RS14075	WP_130171186.1	Na(+)/H(+) antiporter subunit D
	ELS17_RS14105	WP_130171191.1	Na+/H+ antiporter subunit G
	ELS17_RS18175	WP_130171818.1	Na+/H+ antiporter NhaC
	ELS17_RS18315	WP_007107519.1	Na+/H+ antiporter NhaC
	ELS17_RS02745	WP_130170446.1	Na+/H+ antiporter NhaC family protein
	ELS17_RS14030	WP_130171178.1	Na+/H+ antiporter NhaC family protein
	ELS17_RS08810	WP_130170325.1	Na+/H+ antiporter subunit D
	ELS17_RS02765	WP_130169442.1	sodium:calcium antiporter
	ELS17_RS13225	WP_130171051.1	calcium/sodium antiporter
	ELS17_RS13970	WP_130171171.1	calcium/sodium antiporter
	ELS17_RS14080	WP_130171187.1	cation:proton antiporter
	ELS17_RS14090	WP_130171189.1	cation:proton antiporter
	ELS17_RS17095	WP_007107866.1	sodium:calcium antiporter
	ELS17_RS08265	WP_130170248.1	cation:proton antiporter
	ELS17_RS08815	WP_008452176.1	MULTISPECIES: cation:proton antiporter
Compatible solute biosynthesis and uptake	ELS17_RS14425	WP_130171241.1	trehalose-6-phosphate synthase
	ELS17_RS14430	WP_130171242.1	trehalose-phosphatase
	ELS17_RS08295	WP_130170253.1	sodium/proline symporter (OpuE)
	ELS17_RS09955	WP_130170562.1	sodium/proline symporter (OpuE)
	ELS17_RS00335	WP_130169083.1	glutamate-5-semialdehyde dehydrogenase (ProA)
	ELS17_RS00340	WP_007108781.1	glutamate 5-kinase (ProB)
	ELS17_RS00345	WP_081603342.1	pyrroline-5-carboxylate reductase (ProC)
	ELS17_RS02555	WP_130170444.1	glutamate synthase large subunit (gltB, gltD)
	ELS17_RS02650	WP_130169427.1	glutaminase encoded by glsA

Table S5. Putative proteins implicated for UV resistance inferred from genome sequence of *N. altunense* strain 4.1R (SHMR00000000.1)

Nucleotide Excision Repair (NER)	excinuclease ABC subunit (UvrA)	ELS17_RS01125	WP_130169190.1
	excinuclease ABC subunit C (UvrC)	ELS17_RS04460	WP_130169688.1
	excinuclease ABC subunit (UvrB)	ELS17_RS04495	WP_130169694.1
	ATP-dependent DNA helicase (UvrD	ELS17_RS05460	WP_130169845.1
	ATP-dependent DNA helicase (UvrD)	ELS17_RS05655	WP_130169875.1
	ATP-dependent DNA helicase (UvrD)	ELS17_RS05925	WP_130169916.1
	ATP-dependent DNA helicase (UvrD)	ELS17_RS06345	WP_130169982.1
	NAD-dependent DNA ligase (LigA)	ELS17_RS04425	WP_130169683.1
Mismatch Repair System (MRS)	DNA mismatch repair protein (MutS)	ELS17_RS04085	WP_130169634.1
	DNA mismatch repair protein (MutS)	ELS17_RS10335	WP_130170626.1
	DNA mismatch repair endonuclease (MutL)	ELS17_RS04100	WP_130169637.1
	Rec J like exonuclease (Rec J)	ELS17_RS16040	WP_130171492.1
	NAD-dependent DNA ligase (LigA)	ELS17_RS04425	WP_130169683.1
	ATP-dependent DNA helicase (UVRD)	ELS17_RS05460	WP_130169845.1
	ATP-dependent DNA helicase (UVRD)	ELS17_RS05655	WP_130169875.1
	ATP-dependent DNA helicase (UVRD)	ELS17_RS05925	WP_130169916.1
	ATP-dependent DNA helicase (UVRD)	ELS17_RS06345	WP_130169982.1
	DNA mismatch repair protein (MutH)	ELS17_RS13910	WP_130171160.1
	DNA repair exonuclease	ELS17_RS04705	WP_130169728.1
Recombination repair	Rec J like exonuclease	ELS17_RS16040	WP_130171492.1
	recombinase RecA	ELS17_RS02535	WP_130169409.1
	recombinase RecA	ELS17_RS12345	WP_130170917.1
	RecJ like exonuclease	ELS17_RS16040	WP_130171492.1
	DNA double-strand break repair ATPase (Rad50)	ELS17_RS03210	WP_130169508.1
	DNA double-strand break repair nuclease (NurA)	ELS17_RS04715	WP_130169730.1
	DNA double-strand break repair nuclease (NurA)	ELS17_RS17145	WP_007107857.1
	DNA repair and recombination protein (RadA)	ELS17_RS05980	WP_007110149.1
	DNA repair and recombination protein (RadB)	ELS17_RS14985	WP_130171322.1
	Holliday junction DNA helicase (HjC)		
	DNA double-strand break repair protein (Mre11)	fig|1227494.5.peg.724	fig|1227494.5.peg.724
	Bipolar DNA helicase (HerA)	fig|1227494.5.peg.3848	fig|1227494.5.peg.724
	recombinase RecA	WP_130169409.1	ELS17_RS02535
	recombinase RecA	WP_130170917.1	ELS17_RS12345
	recombinase XerD	WP_130171185.1	ELS17_RS14070
	recombinase family protein	WP_130170138.1	ELS17_RS07425
	Holliday junction DNA helicase (HjC)	WP_007109905.1	ELS17_RS04815

Table S6: Putative proteins implicated in H_2O_2-induced oxidative stress tolerance inferred from genome sequence of *N. altunense* strain 4.1R (SHMR00000000.1)

Protein Name	Protein product	Locus tag
Superoxide dismutase (SOD)	WP_007109425.1	ELS17_RS16355
Catalase (cat)	WP_130170217.1	ELS17_RS08045
Catalase/peroxidase HPI (katG)	WP_130170850.1	ELS17_RS11890
Thioredoxin-dependent thiol peroxidase	WP_130171574.1	ELS17_RS16620
Glutathione S-transferase family protein	WP_130171170.1	ELS17_RS13965
Alkyl hydroperoxide reductase (AhpC)	fig|1227494.5.peg.2453	fig|1227494.5.peg.2453
Glutaredoxin	WP_130169813.1	ELS17_RS05270
Glutaredoxin	WP_007109283.1	ELS17_RS13990
Glutaredoxin	WP_007109654.1	ELS17_RS08665
Thiol reductase thioredoxin	WP_007110841.1	ELS17_RS02700
Thioredoxin reductase	WP_130171303.1	ELS17_RS14840
Thioredoxin	WP_130171454.1	ELS17_RS15950
Thioredoxin-dependent thiol peroxidase	WP_130171574.1	ELS17_RS16620
Thioredoxin (trxA)	WP_130170585.1	ELS17_RS10130
Thioredoxin	WP_130170808.1	ELS17_RS11615

A

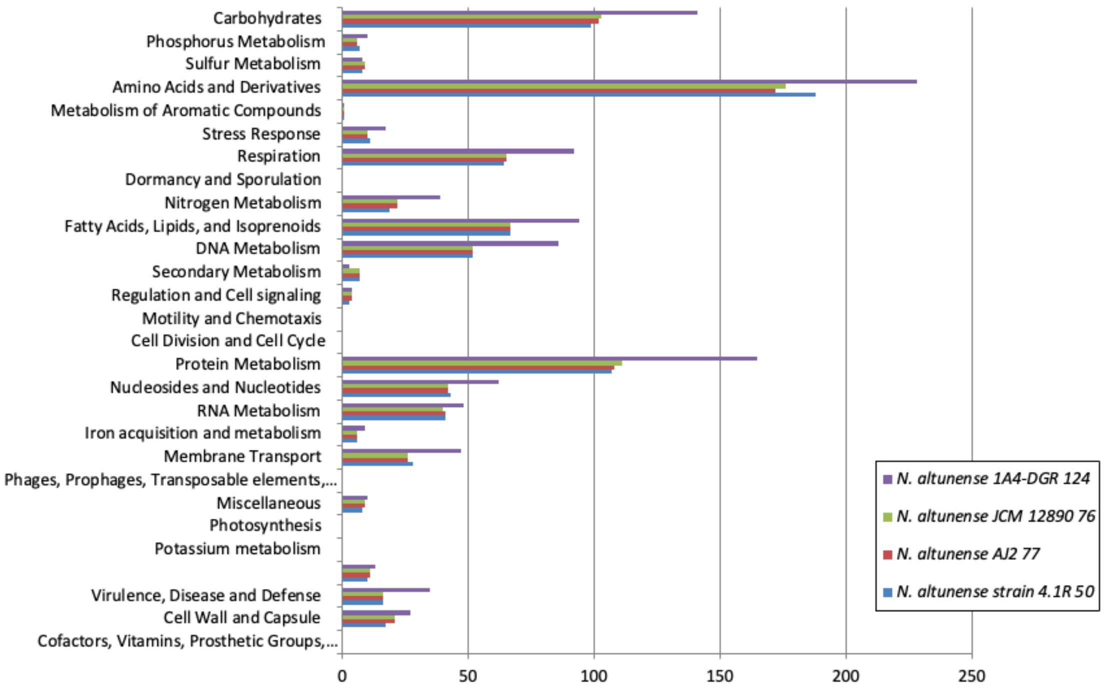

B

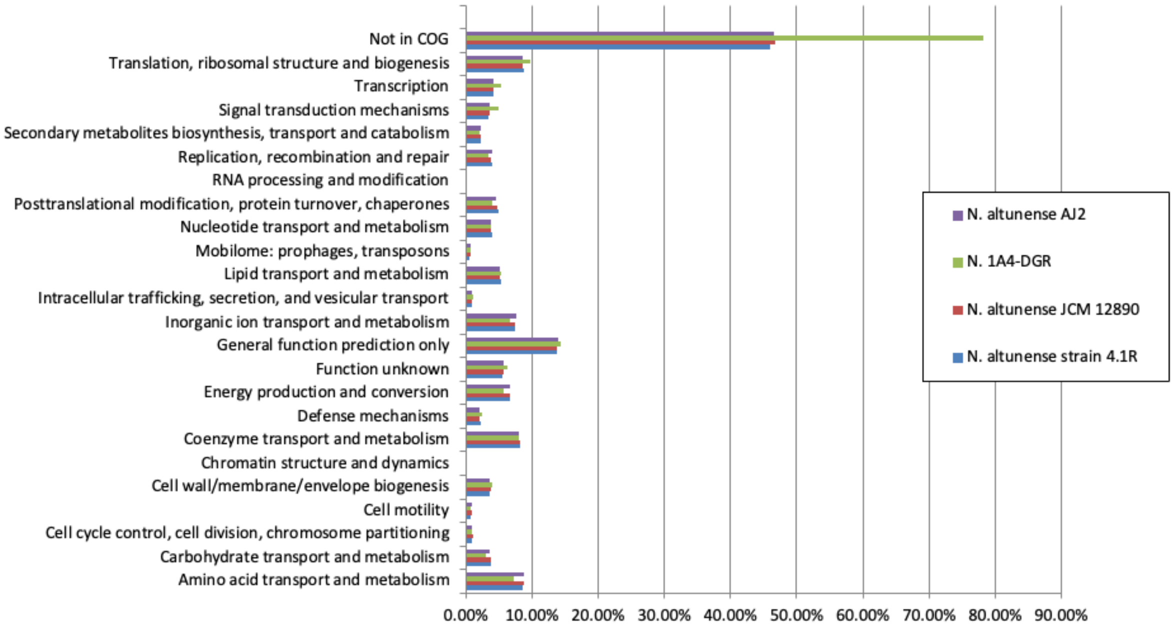

Figure S2. Functional profile
